# Large Language Model-Powered Protected Interface Evasion: Automated Discovery of Broken Access Control Vulnerabilities in Internet of Things Devices

**DOI:** 10.3390/s25092913

**Published:** 2025-05-05

**Authors:** Enze Wang, Wei Xie, Shuhuan Li, Runhao Liu, Yuan Zhou, Zhenhua Wang, Shuoyoucheng Ma, Wantong Yang, Baosheng Wang

**Affiliations:** College of Computer Science and Technology, National University of Defense Technology, No. 137 Yanwachi Street, Changsha 410073, China; wangenze18@nudt.edu.cn (E.W.); lishuhuan21@nudt.edu.cn (S.L.); runhaoliu@nudt.edu.cn (R.L.); yzhou@nudt.edu.cn (Y.Z.); wzh15@nudt.edu.cn (Z.W.); sygfkd@nudt.edu.cn (S.M.); yangwt0805@nudt.edu.cn (W.Y.); bswang@nudt.edu.cn (B.W.)

**Keywords:** protected web interfaces, broken access control, large language model, mutation-based fuzzing, internet of things

## Abstract

Broken access control vulnerabilities pose significant security risks to the protected web interfaces of IoT devices, enabling adversaries to gain unauthorized access to sensitive configurations and even use them as stepping stones for attacking the intranet. Despite its ranking as the first in the latest OWASP Top 10, there remains a lack of effective methodologies to detect these vulnerabilities systematically. We present ACBreaker, a novel methodology powered by a large language model (LLM), to effectively identify broken access control vulnerabilities in the protected web interfaces of IoT devices. Our methodology consists of three stages. The initial stage transforms firmware code that exceeds the LLM context window into semantically intact code snippets. The second stage involves using an LLM to extract device-specific information from firmware code. The final stage integrates this information into the mutation-based fuzzer to improve fuzzing effectiveness and employ differential analysis to identify vulnerabilities. We evaluated ACBreaker across 11 IoT devices, analyzing 1,274,646 lines of code and discovering 39 previously unknown vulnerabilities. We further analyzed these vulnerabilities, categorizing them into three types that contribute to protected interface evasion, and provided mitigation suggestions. These vulnerabilities were responsibly disclosed to vendors, with CVE IDs assigned to those in six IoT devices.

## 1. Introduction

With the rapid development and widespread adoption of Internet of Things (IoT) technology, IoT devices have become crucial to modern network infrastructure. The global number of IoT devices is predicted to increase from 1.88 billion in 2024 to 4 billion by 2030, showing a significant growth trend [1,2]. Among these devices, Embedded Web Applications (EWAs) serve as critical components for IoT device management and configuration. Leading IoT device manufacturers like Netgear, TP-Link, and ASUS have integrated EWAs into their WiFi routers, NAS devices, and other products, providing users with essential functionalities such as device configuration, status monitoring, and firmware updates.

However, the protected interfaces widely implemented in EWAs are susceptible to broken access control vulnerabilities. This security flaw primarily arises because IoT device developers do not strictly adhere to best security practices during development. Consequently, many protected interfaces have flawed access control mechanisms. These flaws allow attackers to access device configuration information and expose sensitive data. Such vulnerabilities can also serve as a stepping stone for subsequent attacks on corporate or home internal networks. Due to their widespread impact and potential for significant harm, broken access control vulnerabilities was ranked first in the latest OWASP Top 10 Web Application Security Risks [3]. Additionally, they were featured in the 2024 CWE Top 25 [4] under two critical categories, ranking 9th and 18th, respectively: CWE-862 (Missing Authorization) [5] and CWE-863 (Incorrect Authorization) [6]. This highlights the increasing prevalence of broken access control vulnerabilities and the critical need to address them.

Previous studies have insufficiently addressed the access control security in protected web interfaces of IoT devices. Hidden interface discovery research, represented by IoTScope [7], primarily focuses on identifying open interfaces accessible without authentication, while tools like Firmalice [8] emphasize detecting hard-coded credentials in firmware. However, these studies fail to systematically investigate protected interfaces that, despite having access control mechanisms, possess implementation flaws. In terms of technical approaches, existing research primarily relies on fuzzing [9,10,11,12,13] and taint analysis [14,15,16]. Fuzzing tools such as Boofuzz [17] are designed to target memory vulnerabilities and struggle to generate effective payloads capable of evading access controls. Although taint analysis is widely employed in vulnerability detection, it faces challenges in access control scenarios, notably in accurately defining relevant taint sources and sinks. Consequently, existing research lacks systematic studies on broken access control in protected interfaces and faces dual technical challenges in code heterogeneity and fuzzing efficiency, resulting in numerous potential vulnerabilities remaining undiscovered.

**Figure 1:**
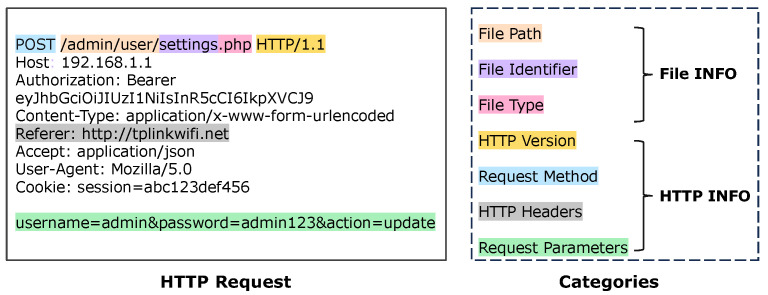
Structure of HTTP Request Components in IoT Web Interface Analysis with Highlighted Feature Categories.

In this paper, we introduce ACBreaker, a novel methodology aimed at addressing this gap. During the development of this framework, we encountered three key challenges: (1) How can we ensure that the LLM receives semantically complete interface code for analysis? Web-related code often exceeds the LLM’s context length. Splitting it based on length would prevent the LLM from analyzing the complete implementation of the interface, potentially resulting in missed vulnerabilities. (2) How can we extract device-specific information from the code to constrain fuzzing boundaries? To effectively discover broken access control vulnerabilities in protected interfaces, we need to extract information from the firmware code. This information is used to construct both the collection of web interfaces for fuzzing and the valid input values required by the mutation operator. However, IoT firmware employs multi-language hybrid development, and interfaces and parameters are often generated dynamically, making it challenging to address using existing static analysis tools. (3) How can we generate mutation requests that trigger broken access control vulnerabilities? As shown in Figure 1, mutation request generation requires performing structured mutations on HTTP requests sent to the protected interface. Only well-structured mutation requests can successfully trigger such vulnerabilities. Additionally, when multiple interfaces share the same access control mechanisms, it is necessary to consider how we can reuse a successful mutation chain to identify all affected interfaces quickly.

Our methodology consists of three stages, each addressing a specific challenge. (1) In the firmware preprocessing stage, we designed a call-relationship-based code-slicing strategy to transform firmware code exceeding the LLM context window into semantically complete code snippets suitable for LLM analysis. (2) In the intelligent firmware analysis stage, we constructed prompts based on a chain-of-thought and employed a two-round inference method to guide the large language model in extracting device-specific information from the firmware code. This information includes paths, parameters, and other elements that can provide valid values for subsequent fuzzing. (3) In the mutation-based fuzzing stage, ACBreaker utilized device-specific information extracted from firmware to generate baseline requests and applied 17 carefully designed mutation operations for structured request mutation. ACBreaker adopts an “effective mutation prioritization” scheduling strategy to fuzzing and utilizes multi-dimensional differential analysis to identify broken access control vulnerabilities effectively.

We implemented a prototype of ACBreaker and evaluated it against 11 IoT devices. The firmware code of these devices contains script files (such as PHP, JavaScript, and Lua) and decompiled binary programs (such as httpd and CGI), totaling 1,274,646 lines. Our evaluation results demonstrate that ACBreaker can effectively detect broken access control vulnerabilities in protected interfaces. ACBreaker discovered 39 previously unknown vulnerabilities, which affect a total of 508 protected interfaces across the evaluated devices. Through analysis, we categorized these vulnerabilities into three primary types of access control evasion: HTTP path manipulation, parameter manipulation, and HTTP header manipulation. We have responsibly reported all discovered vulnerabilities to the affected device manufacturers, with some manufacturers, such as Netgear, TP-Link, D-Link, Redmi, and Xiaomi, having confirmed these security issues and assigned CVE IDs.

In summary, this paper makes the following main contributions:

- *New Approach:* We introduce ACBreaker, a novel LLM-powered methodology to automatically discover broken access control vulnerabilities in protected interfaces of IoT devices. This approach leverages the code comprehension capabilities of the LLM to extract device-specific information from heterogeneous firmware, thereby constraining the fuzzing space. Additionally, ACBreaker employs 17 carefully designed mutation operators to perform structured request mutation and utilizes multi-dimensional differential analysis to effectively identify broken access control vulnerabilities.
- *Real-World Impact:* We evaluated our methodology on 11 IoT devices, analyzing 1 274 646 lines of code, and uncovered 39 previously unknown broken access control vulnerabilities. These vulnerabilities were responsibly reported to the affected vendors, including Netgear, TP-Link, D-Link, Redmi, and Xiaomi, who confirmed the vulnerabilities and assigned CVE IDs to the vulnerabilities found in six IoT devices.
- *New Findings and Mitigation:* We identified three types of access control evasion in protected IoT device web interfaces: (1) HTTP path manipulation, (2) parameter manipulation, and (3) HTTP header manipulation. We also provide mitigation suggestions for addressing each of these evasion techniques.

## 2. Related Work

**IoT Interface Security and Broken Access Control Vulnerabilities.** Existing research on IoT device interface security primarily focuses on discovering and analyzing hidden open interfaces. For instance, Xie et al. [7] utilized lightweight static analysis techniques to automatically detect hidden interfaces in IoT firmware, revealing numerous unauthenticated sensitive interfaces that could lead to configuration and information leakage risks. Subsequent works, such as APIScope [18] and LeakScope [19], respectively, addressed undocumented APIs and data leakage issues. Still, these studies share a common characteristic of focusing on open interfaces that can be accessed without authentication. Unlike previous studies, our research focuses on interfaces that are intended to be protected by access control mechanisms but contain security flaws. These flaws, arising from oversights in policy configuration or code implementation, allow attackers to evade protections and gain unauthorized access.

Previous research on access control vulnerabilities primarily revolves around the security of authentication parameters. Shoshitaishvili et al. [8] introduce an authentication bypass detection model based on input determinism and developed the Firmalice framework based on symbolic execution technology, achieving automated detection of hardcoded credentials. Huang et al. [20] improved analysis efficiency by introducing a concurrent execution framework based on symbolic execution. Zhang et al. [21] proposed BACDetector and Zuo et al. [22] developed the Autoforge framework, which, respectively, detects authentication flaws in device binding and server-side authentication processes through man-in-the-middle attacks and requests forgery techniques. Additionally, access control flaws in cloud services and cloud interactions [23,24,25] have also garnered some attention. However, these studies generally focus on single-dimensional analysis of authentication parameters, making it difficult to effectively detect broken access control vulnerabilities in protected interfaces caused by complex factors such as HTTP path parsing and request header validation.

Research by Orange Tsai [26] revealed that inconsistencies in parser behavior during path normalization can lead to access control evasion risks and discover multiple path traversal and remote code execution vulnerabilities in real-world applications. This work provided important insights, prompting us to investigate access control mechanisms based on HTTP paths and design-targeted mutation operators accordingly to assist fuzzing in quickly identifying such security issues.

**LLM-Assisted Vulnerability Discovering.** Recent research demonstrates that LLMs exhibit unique technical advantages in code analysis and vulnerability discovery. Ma et al. [27] confirmed that LLMs possess capabilities similar to abstract syntax tree parsers, enabling in-depth semantic analysis of code. This code comprehension capability has strengthened the transition from random mutation to guided mutation in fuzzing. For example, DFUZZ [28] leverages LLM to infer boundary cases from API code and transfer them to the testing of other APIs, while Hu et al. [29] improved the input quality of traditional gray-box fuzzing by introducing a GPT-based seed Mutator. Regarding test case generation, research by Asmita et al. [30] demonstrated that LLMs can efficiently generate target-specific initial seeds, significantly increasing the rate of program crash discoveries. Zhang et al.’s [31] evaluation further showed that, given sufficient context, test cases generated by LLMs outperform those produced by traditional tools. For instance, CrawlMLLM [32] leverages a multimodal LLM-based agent to assist in test case generation by interpreting webpage screenshots and content.

The advantage of LLMs in multi-language code analysis is particularly evident, as LLMs can understand and analyze code written in multiple languages, such as C, PHP, and Lua, without requiring separate feature extraction rules for each language [33,34,35,36,37]. This capability enables LLMs to directly extract key information, such as HTTP request parameters and interface paths, making them ideal for analyzing the heterogeneous code found in IoT firmware. Fuzz4All [38] is an example of how LLMs leverage multi-language understanding to overcome the limitations of traditional tools that are often restricted to a single language.

Based on these strengths in code comprehension and multi-language support, we designed the intelligent firmware analysis stage in ACBreaker. This stage effectively addresses the challenges of heterogeneous code analysis in IoT firmware, enhancing the efficiency of discovering broken access control vulnerabilities by constraining the fuzzing space and extracting device-specific information from the firmware.

## 3. Background and Motivation

### 3.1. Web Interfaces in IoT Devices

Embedded Web Applications (EWAs), serving as critical components for IoT device management, exhibit significant code heterogeneity. At the server level, device manufacturers typically employ lightweight web servers (such as mini_httpd, boa) or proprietary implementations. On the application layer, backend logic may utilize script languages like PHP or CGI programs written in C/C++, while the frontend is typically built using HTML and JavaScript [39,40]. This combination of multiple programming languages and technology stacks significantly increases the difficulty of conducting a systematic analysis of EWAs.

In EWAs, web interfaces act as the interaction bridge between clients and device functionalities [41], typically consisting of HTTP paths, HTTP versions, request methods, parameters, and HTTP headers, as illustrated in Figure 1. These interfaces are central to device management functionality, enabling users to perform device configuration, status monitoring, and other operations. Web interfaces can be categorized into open and protected interfaces based on differences in access control mechanisms. Open interfaces (such as login pages and static resources) allow direct access. In contrast, protected interfaces (such as device configuration and system status queries) require valid access credentials, specific parameters, or additional security mechanisms to ensure authorized access.

This study focuses on detecting broken access control vulnerabilities in protected interfaces of EWAs. These protected interfaces often involve sensitive operations and access to critical information. If the access control mechanisms are flawed, attackers may evade authentication or authorization checks, gaining access to web interfaces that should be protected. This can lead to information leakage or the unauthorized modification of device configurations, among other security risks. Furthermore, such vulnerabilities can serve as stepping stones for exploiting other vulnerabilities, such as command injection [42].

### 3.2. Broken Access Control Vulnerabilities

Access control is a core security mechanism for IoT device web interfaces [43,44], used to determine whether users have permission to access specific resources. In EWA interfaces, this mechanism typically implements authentication and authorization policies based on the HTTP protocol. However, due to the heterogeneity of firmware code and the lack of security awareness among developers, implementing access control mechanisms in IoT devices often exhibits flaws, leading to broken access control vulnerabilities.

Broken access control vulnerabilities primarily manifest as defects in the permission verification logic within the HTTP request processing pipeline. Attackers can evade the protected interfaces by modifying different components of HTTP requests, such as changing HTTP request methods (some interfaces only validate GET requests and neglect POST request validation [45]) or constructing special URL paths (developers might configure certain interface names to evade verification, thereby enabling protected interface evasion, as seen in the case in Section 4.1). These security flaws arise from developers’ failure to comprehensively consider various access scenarios, resulting in oversights in implementing access control mechanisms.

Recent security research has highlighted the severe security risks associated with broken access control vulnerabilities in protected interfaces. For instance, in the CVE-2020-5902 case [46], F5 BIG-IP’s device management interface exhibited a flaw in access control validation, allowing attackers to evade the permission verification mechanism of protected interfaces by constructing special HTTP paths. This enabled them to execute commands or upload malicious files, ultimately gaining complete control of the device. However, existing studies rely on manual analysis to identify vulnerabilities, leading to incomplete detection and inefficiency. Currently, there remains a lack of an automated and efficient method to detect broken access control vulnerabilities in protected interfaces in IoT devices, which drives our research.

## 4. Overview

### 4.1. Threat Model

The access control mechanism for protected interfaces relies on the collaboration between the access control and routing modules. Taking the protected interface “/RST_status.htm” in the Netgear WNR614 device as an example, we analyze the implementation logic and security flaws of its access control mechanism. This interface, which displays device status information (e.g., IP address, firmware version), was extracted from the device’s “setup.cgi” binary component by ACBreaker.

**Figure 2:**
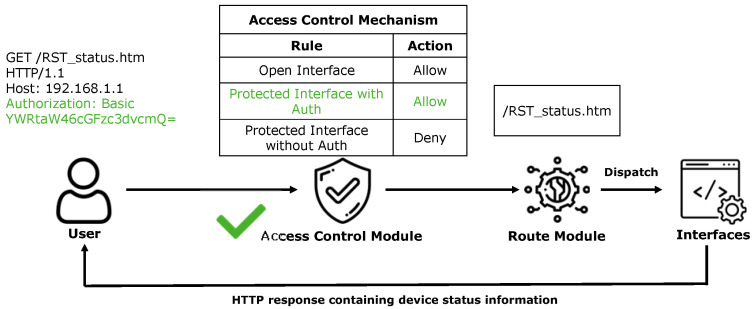
Authenticated access to the protected interface: The access control module validates provided credentials and permits routing to the target resource.

**Figure 3:**
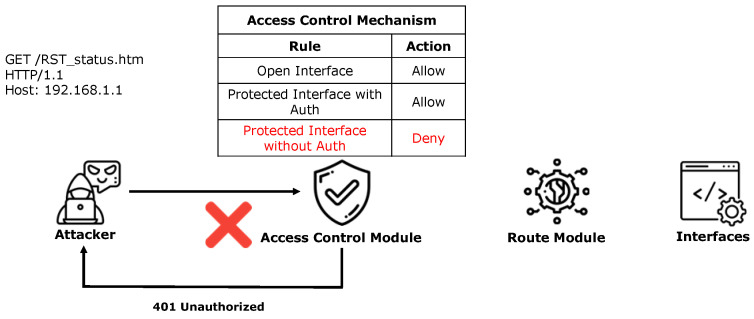
Unauthenticated access to the protected interface: The access control module blocks requests lacking credentials.

In the normal access process (as shown in Figure 2), when users access this interface, they are required to provide authentication credentials (e.g., Authorization: Basic YWRtaW46cGFzc3dvcmQ=). Once the access control module verifies the provided credentials as valid, the routing module forwards the request to the target interface and returns the device status information to the user. Conversely, when an unauthorized attacker attempts to access this interface without valid credentials (as shown in Figure 3), the access control module denies access, thereby protecting the interface.

**Figure 4:**
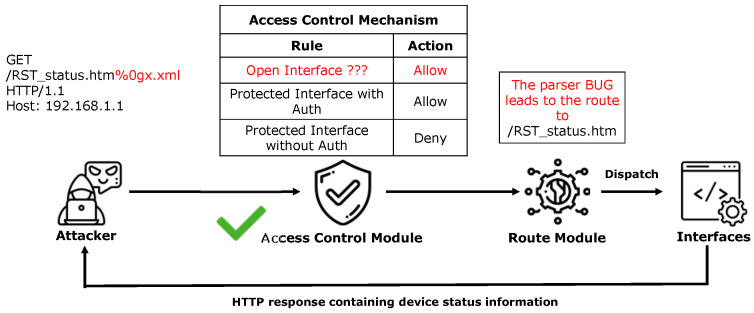
Access control evasion for the protected interface: The mutation request exploits parsing divergence between access control and routing modules to reach protectedinterfaces.

However, ACBreaker discovered a critical vulnerability that allows attackers to evade this access control mechanism (as shown in Figure 4). To exploit this vulnerability, the attacker only needs to append a specific character sequence to the URL path (such as “/RST_status.htm%0gx.xml”). This occurs because the access control module determines an interface’s type solely by examining the suffix of its name. As a result, if an attacker modifies a request so that its target interface appears to end with “.xml”, the module mistakenly classifies it as an open interface. Simultaneously, the routing module ignores content after “%0g” and forwards the requests to the protected interface “/RST_status.htm”. As a result, attackers can access this interface and obtain device status information without providing valid authentication credentials.

**Figure 5:**
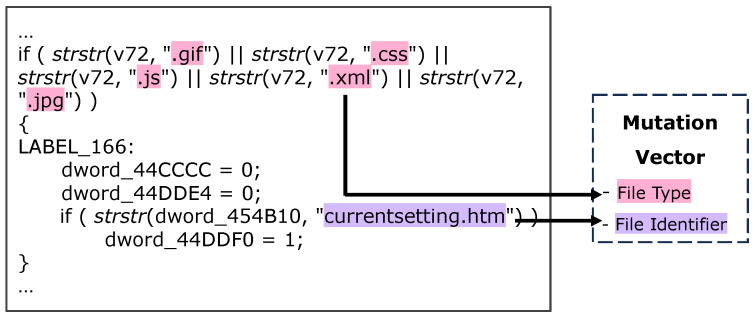
Defective access control mechanism code snippet in Netgear’s WNR614 device, along with its mutation vectors.

Analyzing the device firmware code (see Figure 5), we identified that the root cause of this vulnerability is a critical flaw in implementing the access control logic. The access control module only identifies protected interfaces through suffix matching (e.g., “.xml”, “.gif”, “.js”) or file identifier matching (e.g., “currentsetting.htm”). This string-based access control strategy introduces security risks due to inconsistent URL parsing logic between the access control and routing modules. Such implementation flaws allow attackers to gain unauthorized access to critical configuration privileges (e.g., network settings and DNS configurations). More importantly, since IoT devices are typically deployed at network boundaries, these vulnerabilities may serve as entry points for attackers to penetrate internal networks.

### 4.2. Challenges in Discovering Broken Access Control Vulnerabilities

Given the serious security threat posed by broken access control vulnerabilities, we aim to develop an effective methodology to identify them in protected interfaces. To achieve this goal, we face three key challenges.

**Challenge 1: How can we ensure that the LLM receives semantically complete interface code for analysis?**

Web-related code files (such as “httpd.decompiled.c” obtained from decompiling “httpd”) typically contain the processing logic of multiple interfaces, and their file sizes often exceed the LLM’s context length (128 K tokens). To enable the LLM to analyze access control mechanism implementations within these interfaces, we need to extract complete processing logic for each interface from these large files. However, this extraction is not as simple as splitting code by length, as interface implementations often involve call relationships between multiple functions. Simple segmentation would break these call relationships, preventing the LLM from understanding the complete interface processing flow and potentially missing access control vulnerabilities.

**Challenge 2: How can we extract device-specific information to constrain fuzzing boundaries?**

In detecting broken access control vulnerabilities in protected interfaces, a lack of in-depth understanding of the code implementation can lead the fuzzer to blindly search within a vast input space, making it difficult to discover vulnerabilities efficiently. To accurately define fuzzing boundaries, we need to extract the interface and valid value information from the code. However, this task faces significant challenges: the large volume of firmware code makes comprehensive manual analysis impractical; IoT firmware employs multi-language hybrid development (e.g., C, PHP, Lua); and interfaces and parameters are often generated through dynamic string concatenation or complex code logic.

**Challenge 3: How can we generate effective mutation requests to trigger broken access control vulnerabilities?**

The mutation requests that trigger broken access control vulnerabilities in protected interfaces must adhere to the structural specifications of HTTP requests while also being capable of evading the access control mechanism. Since multiple interfaces may reuse the same access control mechanism, these mutation requests must be designed to facilitate the reuse of successful mutation chains through fuzzing scheduling. However, existing tools lack mutation strategies specifically tailored for access control evasion, making it difficult to generate effective mutation requests that both comply with HTTP specifications and successfully evade access control mechanisms.

### 4.3. Our Solution

**Figure 6:**
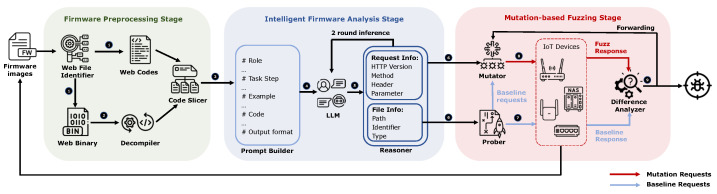
ACBreaker workflow: From firmware preprocessing for LLM analysis, through intelligent firmware analysis with two-round inference, to mutation-based fuzzing with differential analysis for vulnerability detection (red arrows indicate mutation requests, while blue arrows represent baseline requests).

In light of these challenges, we propose an automated methodology called ACBreaker to discover broken access control vulnerabilities in protected interfaces. Figure 6 illustrates an overview of ACBreaker. ACBreaker takes IoT device firmware as input and outputs reports on discovered broken access control vulnerabilities. The workflow of ACBreaker is as follows.

**Solution for Challenge 1: Firmware Preprocessing Stage**

We designed a call-relationship-based firmware preprocessing method to transform firmware code exceeding the LLM’s context length into semantically complete code snippets. The workflow for this stage consists of the following: ① *Web File Identifier*, which processes the decompressed firmware files to identify web-related code, including script files (PHP, HTML, JavaScript, Lua) and binary (httpd, CGI programs); ② *Decompiler*, which converts binary files in web-related files into pseudo-C code, enabling the LLM to understand program logic and control flow; and ③ *Code Slicer*, which employs a call-relationship-based strategy to handle code files exceeding 100K tokens. We observed that web interface processing follows a fixed sequence of function calls, sequentially performing interface registration, parameter parsing, and access control verification. Based on this observation, the code slicer analyzes function call relationships to integrate relevant code into minimal units with complete semantics, thereby satisfying the LLM’s context limitations.

**Solution for Challenge 2: Intelligent Firmware Analysis Stage**

The intelligent firmware analysis stage leverages LLM’s code comprehension capabilities to extract the file and HTTP information from firmware, thereby constraining fuzzing boundaries to enhance fuzzing effectiveness. The workflow for this stage is as follows: ④ *Prompt Builder* constructs specialized prompts for file information and HTTP information using four strategies: role definition, chain-of-thought, few-shot learning, and structured output. ⑤ The *LLM* analyzes the code based on the constructed prompts, generating two types of structured information: *file information* (including paths, file identifiers, and extensions) for constructing interfaces and *HTTP information* (including protocol versions, request methods, headers, and parameters) for guiding mutation. ⑥ *Reasoner* employs a two-round inference strategy to ensure information extraction completeness. The first round focuses on identifying key structural elements of the code and uses placeholders for complex dynamic items. The second round performs an in-depth analysis of the placeholders, restoring parameter constraints and inferring specific instance values. Finally, the *Reasoner* outputs structured analysis results for subsequent fuzzing.

**Solution for Challenge 3: Mutation-based Fuzzing Stage**

In this stage, we designed a mutation-based fuzzing framework to detect broken access control vulnerabilities. The framework includes 17 mutation operators and employs “mutation chain coordination” and “effective mutation prioritization” algorithms to enhance testing depth and breadth. The workflow of this stage is as follows: ⑦ *Prober* conducts web interface probing. It identifies valid interfaces (response code not 404) by sending unauthenticated HTTP requests and records their baseline responses for subsequent differential analysis. ⑧ *Mutator* implements 17 mutation operators across four dimensions: request line, headers, body, and encoding methods. It constructs mutation requests based on information extracted from firmware and dynamically adjusts priorities according to the effective mutation prioritization strategy, ensuring efficient utilization of fuzzing resources. ⑨ *Difference Analyzer* detects vulnerabilities by comparing baseline requests with mutated requests. When an effective mutation operator is discovered, it generates vulnerability reports and feeds the operator back to the *Mutator*. The *Mutator* then prioritizes using proven effective mutation operators to fuzz all pending interfaces, thereby improving vulnerability discovery efficiency.

## 5. ACBreaker

### 5.1. Intelligent Firmware Analysis

**Figure 7:**
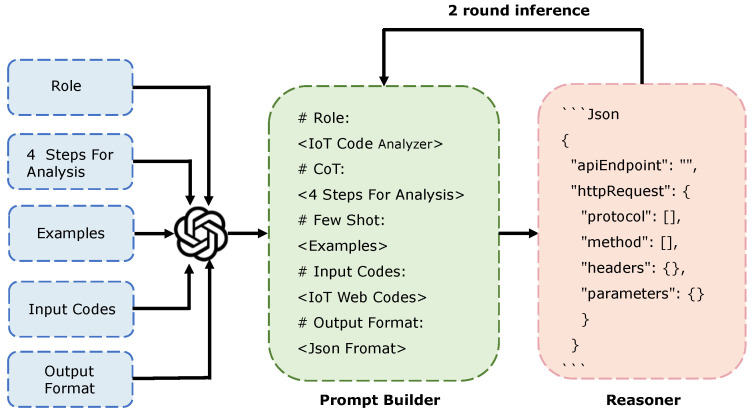
Prompt construction and inference process (using HTTP information as an example).

In the firmware preprocessing stage, we extracted 1,274,646 lines of web-related code from 11 IoT device firmware images, including script files (PHP, JavaScript, Lua, etc.) and decompiled binaries (HTTP servers, CGI programs, etc.). We then employed a code slicer to transform these into semantically complete code snippets that comply with the LLM’s context length limitations. In this stage, we developed an intelligent firmware analysis technique based on the code-understanding and -reasoning capabilities of the LLM, as illustrated in Figure 7. This technique employs a chain-of-thought method to construct analysis prompts and utilizes a two-round LLM inference mechanism to extract key information from heterogeneous code: In the first round, the LLM identifies the structural elements for information extraction and marks portions requiring in-depth analysis. In contrast, it infers the specific constraints and values of these marked items in the second round. The final output includes file information of web interfaces and valid input values, which guide subsequent fuzzing. The following figure details our approach.

#### 5.1.1. Information Extraction

As shown in Figure 1, we extract two categories of key information from the IoT firmware code: file information and HTTP information. This categorization enables the model to focus on extraction tasks in specific dimensions, providing valid input values for subsequent fuzzing, thereby enhancing fuzzing effectiveness.

In the file information dimension, we extract three elements for constructing web interfaces: file path (e.g., “/admin/user/”) specifies the interface access path, file identifier (e.g., “settings”) determines the specific interface, and file type (e.g., “.php”) determines the processing method. In the HTTP information dimension, we extract four elements required for constructing requests: HTTP version and request method are used for constructing the request line; HTTP headers (such as Content-Type, Authorization, etc.) are used to control the behavior of request processing; and request parameters (such as “username=admin&password=admin123”) contain specific values required for business operators.

#### 5.1.2. Four Steps for Analysis

Extracting device-specific information from firmware is a complex analytical task. We decompose it into four steps and employ chain-of-thought prompting to guide the LLM in a step-by-step analysis, thereby enhancing accuracy. This method applies to the extraction of both file information and HTTP information. This section provides a detailed explanation of these four steps using HTTP information extraction as an example.

**Step 1: Programming Language Identification.** T firmware typically employs multi-language hybrid development, including scripting languages such as PHP, JavaScript, Lua, and compiled languages like C/C++. Each language has its specific patterns for handling web interfaces, such as PHP using superglobal variables ($_GET, $_POST) for HTTP request handling, while C/C++ relies on structures and function parameters. We first instruct the LLM to accurately identify the language type of the code being analyzed, enabling it to tailor its analysis strategy accordingly and improve extraction accuracy.

**Step 2: HTTP Request Information Extraction.** In this step, we instruct the LLM to identify HTTP protocol information, primarily including (1) protocol version identifiers (such as HTTP/1.0, HTTP/1.1), (2) HTTP request method declarations, and (3) HTTP header information. To enhance analysis quality, we provide typical HTTP information definition examples, such as headers = {“Content-Type”: “application/json”}, to help the model better recognize this information.

**Step 3: Parameter Analysis.** Parameter analysis comprises three key tasks: (1) Conditional statement analysis; this involves extracting parameter value range constraints by parsing control structures (such as if-else, switch-case) and generating values covering different execution paths, for example, after extracting constraints from “if ($RoleID < 0)”, thereby generating values -1 and 1 to cover different branches. (2) Default value extraction; this comprises obtaining parameter initialization or default values based on variable definitions. (3) Constraint analysis; this consists of generating feasible values that satisfy all explicit constraint conditions related to the parameters.

**Step 4: Dynamic Value Generation.** Dynamic value generation specifically handles parameter values that require computation, primarily involving the generation of session tokens, timestamps, and cryptographic hashes. The LLM infers values that meet constraints by analyzing value generation algorithms (such as specific hash functions) and usage scenarios (such as time format requirements). For instance, for MD5-based token generation logic, the LLM can derive hash values in the correct format.

Finally, we require the LLM to combine the analysis results from the above four steps to provide the final structured information. This multi-step chain-of-thought analysis enables us to obtain accurate and complete file and HTTP information, providing effective values for subsequent mutation-based fuzzing.

#### 5.1.3. Prompt Builder

The design of prompts directly determines the quality of the answer output by the LLM, so we carefully constructed a structured prompt template consisting of five core components to enhance understanding, as shown in Figure 7.

**Role Definition:** We instruct the LLM as an “IoT firmware code analysis expert”. This explicit role constraint enables the LLM to maintain a professional perspective and focus on analyzing IoT device code.

**Analysis Steps:** We begin by instructing the LLM to reason step by step using a chain-of-thought approach [47]. Then, we briefly list and explain the four analytical steps described in Section 5.1.2, accompanied by illustrative examples.

**Examples:** We employ a few-shot learning method, manually designing three real-world examples: HTTP request analysis, parameter analysis, and dynamic value inference. Each example includes input code and corresponding analysis results in JSON format, guiding the LLM in understanding the analysis task and standardizing the output format through specific examples.

**Input Code:** We use XML tags to encapsulate the code obtained from the firmware preprocessing stage for the LLM to analyze.

**Output Format:** We instruct the LLM to output final results in a structured JSON format to ensure result parsability.

We combine the above five components—role definition, analysis steps, few-shot examples, input code, and output format—into a single structured prompt. This complete prompt is submitted as a whole to the LLM, rather than in separate parts, to ensure contextual consistency. We regenerate the complete prompt for each new code analysis task by replacing the “Input Code” component with the new code snippet while keeping the other components unchanged. This ensures that every query provides the LLM with the full analytical context, thereby enhancing extraction accuracy and consistency across multiple tasks.

#### 5.1.4. Reasoner

We designed a Reasoner using a two-round inference strategy to overcome the limitations of single-round LLM analysis, particularly when handling dynamically generated values and complex conditional constraints.

In the first round, using a breadth-first strategy [48], the LLM systematically extracts basic information from the firmware code, including interface paths and HTTP basic parameters. When it encounters complex parameters that require in-depth analysis—such as values derived from encryption functions or conditional logic—it marks them with placeholders or natural language descriptions for further inference.

The second round of inference focuses on processing these marked items. The Reasoner identifies the fields to be analyzed by parsing the JSON output from the first round and performs targeted reasoning to infer valid input values. For example, when analyzing a Lua script that dynamically constructs a parameter like md5_param = md5(seed ...), the Reasoner recognizes the MD5-based generation logic and infers a valid hash-formatted value to satisfy the request constraint. These in-depth analysis results provide valid input values for subsequent fuzzing and are crucial for triggering access control evasion that depends on correctly formatted dynamic inputs.

### 5.2. Mutation-Based Fuzzing

ACBreaker employs mutation-based fuzzing to detect broken access control vulnerabilities in protected interfaces. This approach performs mutations on HTTP requests while preserving their semantic integrity, aiming to evade access control mechanisms and obtain unauthorized access. To achieve this, we designed a fuzzing framework that integrates mutation chain coordination and effective mutation prioritization strategy, as shown in Algorithm 1.
**Algorithm 1** Mutation-based Fuzzing**Require:**  1:  FileINFO: {Path Information, File Identifier, File Type}  2:  HTTPINFO: {Protocol Information, Request Methods, HTTP Headers, Request Parameters}**Ensure:**  3:  Vulnerability Report: {Interface of evasion, Mutation Chain, Mutation Request, Mutation Response}  4:  **function**
Fuzzing(FileINFO, HTTPINFO)  5:  interfaces←∅▹ Initialize interface set  6:  mutations←∅▹ Initialize mutation set  7:  vulnReport←∅▹ Initialize vulnerability report  8:  priorityQueue←∅▹ Priority queue for mutation chains  9:  **for all** path∈FileINFO.paths **do**10:     **for all** identifier∈FileINFO.identifiers **do**11:     **for all** filetype∈FileINFO.types **do**12:        interface←
CombineInterface(path, identifier, filetype)13:        initialReq←
ConstructRequest(interface)14:        initialResp←
SendRequest(initialReq)15:        **if** initialResp.statusCode≠404 **then**16:         baselineReq←initialReq17:         baselineResp←initialResp18:         interfaces.add({interface,baselineReq,baselineResp})19:         **if** baselineResp.statusCode=200 **then**20:           mutations.extend(ExtractMutationInfo(interface))21:         **end if**22:        **end if**23:       **end for**24:     **end for**25:  **end for**26:  mutations←
InitializeMutators(FileINFO, HTTPINFO)27:  pendingInterfaces←interfaces▹ Initialize pending interface set28:  **for all** interface∈interfaces **do**29:    chains←
GenerateMutationChains(mutations)30:    chains←
RemoveConflicts(chains)31:    **while** ¬chains.empty()∨¬priorityQueue.empty() **do**32:      **if** priorityQueue.notEmpty() **then**33:       chain←priorityQueue.pop()34:       pendingInterfaces←interfaces▹ Reset pending interfaces35:       **while** ¬pendingInterfaces.empty() **do**36:       targetInterface←pendingInterfaces.pop()37:       mutatedReq←
ApplyMutationChain(targetInterface.req, chain)38:       mutatedResp←
SendRequest(mutatedReq)39:       result←
DifferenceAnalyzer(targetInterface.resp, mutatedResp)40:       **if** result.isVulnerable **then**41:          vulnReport.add({targetInterface,chain,mutatedReq,mutatedResp})42:       **end if**43:       **end while**44:    **else**45:       chain←chains.pop()46:       mutatedReq←
ApplyMutationChain(interface.req, chain)47:       mutatedResp←
SendRequest(mutatedReq)48:       result←
DifferenceAnalyzer(interface.resp, mutatedResp)49:       **if** result.isVulnerable **then**50:       vulnReport.add({interface,chain,mutatedReq,mutatedResp})51:       priorityQueue.add(chain)▹ Prioritize effective chain52:       **end if**53:    **end if**54:   **end while**55:  **end for**     **return** vulnReport56:  **end function**

The algorithm accepts two inputs: (1) *FileINFO*, containing path information, file identifiers, and file types extracted from firmware, and (2) *HTTPINFO*, containing protocol information, request methods, HTTP headers, and request parameters. The algorithm outputs vulnerability reports documenting discovered broken access control vulnerabilities, including protected interfaces of evasion, successful mutation chains, mutation requests, and the corresponding responses. Its execution process consists of three main phases:

Phase 1: Interface Detection (Lines 5–25). ACBreaker first constructs web interfaces by combining path information, file identifiers, and file types from *FileINFO*. It sends initial HTTP requests to each interface, storing interfaces with response status codes other than 404 and corresponding HTTP response information as baseline data in the interface set for differential analysis. Notably, when the baseline response status code is 200, ACBreaker extracts path and file identifier information from that interface through the *ExtractMutationInfo* function, which mutation operators will use.

Phase 2: Mutation Generation and Execution (Lines 26–47). ACBreaker first initializes the mutation operator set by invoking the *InitializeMutators* function based on *FileINFO* and *HTTPINFO*, and maintains the *pendingInterfaces* set to track interfaces pending testing. For each valid interface, the *GenerateMutationChains* function generates mutation chain coordination, and the *RemoveConflicts* function subsequently removes conflicting mutation chains. During fuzzing, ACBreaker employs an “effective mutation prioritization” scheduling strategy. When a mutation chain successfully triggers a vulnerability, it is immediately added to the *priorityQueue*, and the current fuzz sequence is paused to apply this mutation chain to all interfaces in *pendingInterfaces*.

Phase 3: Differential Analysis. The *DifferenceAnalyzer* function (show in Algorithm 2) analyzes mutation responses through a three-layer filtering mechanism: first filtering invalid responses, then checking for significant status code changes (e.g., from 401/403 to 200/202), and finally verifying whether access control has been successfully evaded through response body analysis.

The following sections will detail the specific implementation of mutation algorithms, mutation operators, and differential analysis strategies.

#### 5.2.1. Mutation Algorithm

We designed a mutation algorithm that integrates mutation chain coordination and effective mutation prioritization to enhance the efficiency of discovering broken access control vulnerabilities.

**Mutation Chain Coordination.** The mutation chain coordination strategy generates fuzzing sequences by combining multiple mutation operators. For each baseline request, the Mutator constructs a complete list of mutation chains in the form of {M1, M2, M3, M1M2, M1M3, M2M3, M1M2M3…}, where each element represents a mutation chain, consisting of a specific combination of mutation operators. To optimize the generation and execution efficiency of mutation chains, this strategy implements two key dynamic optimization mechanisms: (1) *Conflict Detection*: analyzes interactions between operators within mutation chains and automatically removes conflicting combinations, such as those that mutually modify the same HTTP header field; and the (2) *Minimization Principle*: when a shorter mutation chain successfully triggers a vulnerability, ACBreaker automatically eliminates all longer mutation chains containing this successful chain to ensure the simplest vulnerability triggering conditions.

**Effective Mutation Prioritization.** The effective mutation prioritization strategy optimizes fuzzing sequences through dynamic scheduling. The Mutator suspends the current fuzzing sequence when the differential analyzer confirms that a mutation chain successfully evades access control for a protected interface. It prioritizes testing all pending interfaces using this effective mutation chain. This strategy is based on the observation that different web interfaces within the same device often implement similar access control mechanisms, thereby accelerating vulnerability discovery by reusing verified effective mutation chains.

#### 5.2.2. Mutation Operators

The design of mutation operators directly affects the effectiveness and efficiency of discovering broken access control vulnerabilities. We developed a systematic set of mutation operators based on the key components of HTTP requests to create a fuzzer that effectively identifies vulnerabilities in protected interfaces. These mutation operators are categorized into four dimensions: request line (M1–M8), headers (M9–M11), body (M12), and byte level (M13–M17). As shown in Table 1, we designed 17 mutation operators, 12 of which are novel methods introduced in this study (operators not marked with an asterisk). These operators systematically uncover security flaws in protected interfaces, enabling us to identify and classify three common types of broken access control vulnerabilities: path manipulation, header manipulation, and parameter manipulation.

**Request Line Mutation Operators** explore access control evasion based on HTTP request lines. M1 and M2 implement basic mutations of HTTP request methods and protocol versions, respectively. M3 to M5 focus on path mutation designed based on URL parsing features from RFC 3986, including Matrix Parameters (such as transforming /admin/ to /;/admin/), suffix addition, and path hierarchy insertion. M6 and M7 are designed based on relative URI parsing rules from RFC 3986 [49], employing directory traversal sequences (e.g., /static/../admin) to test the server’s path normalization handling. These fuzzing sequences are constructed by combining path and file identifiers through the *ExtractMutationInfo* function. M8 specifically leverages ASP.NET’s cookieless feature for fuzzing.

**Header Mutation Operators** explore access control evasion based on HTTP headers. M9 tests source address verification by injecting IP-related headers (such as X-Remote-IP: 127.0.0.1). M10 probes parsing vulnerabilities by adding URL rewriting-related headers. M11 constructs requests using header information extracted from firmware (e.g., specific Referer values).

**Body Mutation Operators** explore access control evasion based on parameter processing logic. They extract parameter names and values from firmware to create valid parameter combinations, thereby identifying flaws in parameter validation.

**Byte-level Mutation Operators** provide more fine-grained request modifications. These include special character injection (M13), case conversion (M14), Unicode homograph replacement (M15), URL encoding (M16), and control character replacement (M17). These low-level operations probe for parsing implementation flaws by altering character encoding and representation methods, such as mutating /system.php?public.cgi to /system.php%00public.cgi to test for parameter delimiter processing flaws.

In summary, the choice of mutation operators is aimed at simulating a wide range of potential evasion techniques based on attack patterns observed in real-world scenarios, such as the CVE-2024-0204 vulnerability found in Fortra GoAnywhere MFT. This vulnerability exploits a flaw in a URL path parser, allowing an unauthenticated attacker to bypass authentication by manipulating the request path and access interfaces that are supposed to be protected, such as those for creating administrator accounts.

#### 5.2.3. Difference Analyzer

The Difference Analyzer identifies broken access control vulnerabilities by comparing baseline and mutation responses. As shown in Algorithm 2, we designed three complementary detection strategies to accurately capture protected interface evasion characteristics: preprocessing strategy, status code transition-based detection, and response body difference-based detection.
**Algorithm 2** Difference Analysis for Vulnerability Detection**Require:**  1:  baselineResp: Response from baseline request  2:  mutResp: Response from mutation request  3:  hashQueue: A queue or dictionary to store the frequency of response body hashes**Ensure:**  4:  isVulnerable: Boolean indicating if a vulnerability is detected  5:  **function** DifferenceAnalyzer(baselineResp, mutResp, hashQueue)  6:  **if** mutResp.statusCode∈{0,404,501,400} **or** mutResp.body=∅ **then return** {isVulnerable:false}  7:  **end if**  8:  **if** baselineResp.body=mutResp.body **then return** {isVulnerable:false}  9:  **end if**10:  **if** baselineResp.statusCode∈{401,403} **and** mutResp.statusCode∈{200,202} **then**11:     hash←
FNV1aHash(mutResp.body)12:     frequency←
GetHashFrequency(hash, hashQueue)13:     **if** frequency>5 **then**    **return** {isVulnerable:false}14:     **end if**15:     staticContent←
FilterDynamicContent(mutResp.body)16:     similarity←
CalculateSimilarity(baselineResp.body, staticContent)17:     **if** similarity<0.9 **then**    **return** {isVulnerable:true}18:     **end if**19:  **end if**    **return** {isVulnerable:false}20:  **end function**

**Preprocessing Strategy (Lines 5–8).** This strategy first filters out invalid responses, including responses with status codes 400, 404, 501, and empty response bodies. Subsequently, ACBreaker compares the content of mutation response bodies with baseline response bodies. Even if the status code changes, identical response content generally indicates that the server’s error-handling mechanism was activated, rather than a successful evasion of the protected interface. For example, we often encounter cases where mutating the request method to HEAD results in a 200 status code but with an empty response body. **Status Code Transition-based Detection Strategy (Line 10).** This strategy determines whether access permissions have changed by alterations in response status codes. When protected interface evasion is successful, status codes typically transition from 401 (Unauthorized) or 403 (Forbidden), indicating restricted access, to 200 (OK) or 202 (Accepted), indicating successful requests. ACBreaker identifies potentially protected interface evasion by monitoring these characteristic status code transitions.

**Response Body Difference-based Detection Strategy (Lines 11–18).** This strategy employs two complementary techniques: (1) Page similarity analysis based on hash value statistics uses the FNV-1a hashing algorithm to calculate feature values of the response body and filters out frequently occurring non-sensitive page content, such as login-redirect pages or generic error messages, through a frequency threshold, and (2) page comparison with dynamic content filtering identifies and filters dynamic content in responses (such as timestamps, session IDs) through the *FilterDynamicContent* function, then calculates the similarity of static content. When similarity falls below 0.9 (this threshold is set to minimize errors in identifying different strings as identical and maintain consistency with existing SOTA research [50]), the strategy determines whether there is a valid protected interface evasion.

### 5.3. ACBreaker Prototype

We implemented the ACBreaker prototype to discover access control vulnerabilities in protected interfaces automatically. ACBreaker uses a modular architecture designed for seamless integration with existing security tools and frameworks. Written in Python, it comprises 11,990 lines of code and covers the entire workflow from firmware analysis to vulnerability detection.

In the firmware preprocessing stage, ACBreaker integrates three core modules. The Web File Identifier uses regular expressions and heuristic rules to detect web-related files in the firmware. It can work with existing firmware analysis tools, such as Binwalk and Firmadyne, for firmware extraction and analysis. The decompiler converts binary files into pseudo-C code and can interface with existing decompilation tools like Ghidra and IDA Pro to enhance the accuracy and readability of decompilation outputs. The code slicer analyzes call relationships, breaking down code files with over 100 K tokens into semantic blocks, ensuring compatibility with large language models (LLMs).

In the intelligent firmware analysis stage, ACBreaker uses LangChain, which includes a prompt builder and a Reasoner. The prompt builder creates prompt templates for information extraction by combining role definitions, chain-of-thought strategies, and few-shot learning. The Reasoner employs two rounds of reasoning to ensure the completeness and accuracy of the extracted information. LangChain integrates with other AI-based models, such as OpenAI’s GPT models or Google’s Gemini, enhancing the system’s information extraction capabilities. Users can easily connect their own models via API, ensuring flexibility and scalability.

In the fuzzing stage, ACBreaker operates through three collaborative modules: the Prober, the Mutator, and the Difference Analyzer. The Prober uses the requests [51] library to identify valid web interfaces. The Mutator generates mutation requests based on 17 specialized mutation operators to fuzz-protected interfaces. The differential analyzer examines baseline requests and mutated requests to detect vulnerabilities. All request data are managed using an SQLite database. All components communicate via JSON, maintaining a consistent and coherent workflow.

The modular design and open interfaces of ACBreaker ensure its adaptability and ease of integration into different environments, enhancing reproducibility by allowing users to customize components and workflows based on their specific needs.

## 6. Evaluation

### 6.1. Experimental Setup

**Dataset.** We evaluated ACBreaker on 11 commercial IoT devices from leading manufacturers, including Netgear, TP-Link, D-Link, Xiaomi, and ASUS. All evaluations were conducted on real physical devices rather than simulated environments. As shown in Table 2, these IoT devices exhibit heterogeneous web architectures. Their web servers are primarily implemented using lightweight solutions such as “cgibin” and “minihttpd” while the applications combine multiple technology stacks (e.g., binary, PHP, Lua, ASP, and HTML), demonstrating the inherent heterogeneity of EWAs. The diversity and complexity of these technology stacks provide a comprehensive testing dataset for assessing the effectiveness of ACBreaker in real-world scenarios.

**SOTA solutions for comparison.** We compared ACBreaker with the state-of-the-art open-source tools. Nomore403 [52] is a popular open-source tool on GitHub for detecting broken access control vulnerabilities. It has garnered over 1.2 k stars and represents state-of-the-art rule-based vulnerability detection. Boofuzz [17], a widely recognized fuzzer, focuses on network protocol fuzzing. Its robust protocol handling capabilities and sophisticated mutation strategies exemplify best practices for fuzzing IoT devices. We selected these tools because ACBreaker also employs black-box fuzzing techniques during its fuzzing stage, making them suitable for direct comparison.

**Setup.** In this experiment, we deployed two models, Qwen-2.5-Coder-32B [53] and GPT-4o [54], to evaluate the generality of our intelligent firmware analysis approach. Both models were configured with a temperature parameter of 0.2 to ensure consistent output. The open-source Qwen-2.5-Coder-32B was deployed on an Ubuntu 22.04 server equipped with dual NVIDIA A100 GPUs, with its inference speed optimized through the vLLM [55] framework, and it provided services to the analysis module via API. In contrast, the commercial model GPT-4o was accessed via its API. The other modules of ACBreaker, including firmware preprocessing and mutation fuzzing, ran on an Ubuntu 22.04 system with an Intel Core i7 (2.6GHz) processor and 16GB of RAM. The API cost for processing firmware from 11 IoT devices using GPT-4o totaled USD 167. The binary files in the firmware were decompiled using IDA Pro to generate pseudo-C code for subsequent LLM-based analysis. All tested IoT devices were reset to factory settings and initialized to their default configurations prior to experimentation.

### 6.2. Overall Findings

**Results.** After eight hours of fuzzing per device, ACBreaker generated an average of 22,546 mutation requests per IoT device. In total, ACBreaker identified 39 distinct vulnerabilities, each corresponding to a unique payload capable of evading the access control mechanisms of protected interfaces. Notably, one payload can evade multiple interfaces, resulting in 508 affected protected interfaces across the devices. We analyzed each vulnerability and categorized them into three types based on the evasion methods of the access control mechanisms: (1) HTTP path manipulation, (2) parameter manipulation, and (3) HTTP header manipulation. Section 6.5 provides detailed case studies. As shown in Table 3, 10 out of the 11 devices in the dataset had at least one broken access control vulnerability. We have responsibly disclosed these vulnerabilities to the respective manufacturers, and six IoT devices have been assigned CVE IDs.

### 6.3. Comparison with State of the Art

We compared ACBreaker with nomore403 and Boofuzz based on two metrics commonly used in black-box fuzzing studies [56]: (1) the number of vulnerabilities discovered after 8 h of fuzzing and (2) the diversity of vulnerability types identified.

**Configuration.** We accounted for each tool’s unique features in our comparative analysis. Since both nomore403 and Boofuzz require valid interfaces as input, we used baseline requests generated by the Prober as common input data. Moreover, because Boofuzz does not include a vulnerability detector, we incorporated ACBreaker’s Difference Analyzer into it for a fair comparison. We set a 5-min fuzzing limit per interface for all three fuzzing tools, with a total runtime of 8 h, consistent with previous studies [56]. Additionally, we conducted three control experiments: (1) ACBreaker(noalg), which removes the mutation scheduling algorithm but still utilizes the GPT model; (2) ACBreaker(GPT), utilizing the complete GPT model; and (3) ACBreaker(Qwen), employing the complete Qwen model. This ablation study was designed to evaluate the contributions of individual technical components.

**The Number of Vulnerability Discovery Comparison.** We summarize the number of vulnerabilities discovered by each tool in this experiment in Table 4. ACBreaker demonstrated a significant advantage, with ACBreaker(GPT) identifying 39 vulnerabilities affecting 508 protected interfaces and ACBreaker(Qwen) identifying 33 vulnerabilities affecting 498 protected interfaces. Through manual verification, all 39 vulnerabilities detected by ACBreaker were confirmed to be valid and exploitable, with no false positives, thanks to our three complementary detection strategies detailed in Section 5.2.3. In contrast, nomore403 identified only four vulnerabilities affecting four protected interfaces, while boofuzz did not detect any vulnerabilities. Specifically, ACBreaker discovered HTTP path manipulation-type access control evasion in devices 1, 2, and 11, which affected all protected interfaces, highlighting the severity of such access control flaws.

There are two main reasons behind ACBreaker’s superior performance, particularly in detecting 0-day vulnerabilities. First, extracting device-specific information based on LLM enables ACBreaker to accurately retrieve valid values to constrain the fuzzing boundaries, thereby effectively uncovering vulnerabilities. Secondly, ACbreaker’s 17 carefully designed mutation operators and its “mutation chain coordination” strategy can effectively uncover in-depth vulnerabilities that triggered the need for multiple mutation operations. This systematic mutation approach significantly outperforms the seven basic mutation rules of nomore403 and the random mutation strategy of boofuzz.

Furthermore, the ACBreaker(noalg) version, which removes the mutation scheduling algorithm, only discovered 28 vulnerabilities affecting 104 protected interfaces. This substantial performance gap underscores the importance of our proposed “effective mutation prioritization” scheduling algorithm. By reusing successful mutation operators, the algorithm allows for the exploration of a broader range of vulnerabilities within the same timeframe, thereby enhancing the efficiency of vulnerability discovery.

**The Categories of Vulnerability Discovery Comparison.** ACBreaker identified three categories of broken access control vulnerabilities (HTTP path manipulation, parameter manipulation, and HTTP header manipulation), while nomore403 only detected HTTP header manipulation, and boofuzz did not identify any categories. The comprehensive detection capability of ACBreaker is primarily attributed to its systematic mutation operator design. This strategy is based on the fundamental components of HTTP requests and implements mutations across four dimensions: request line, headers, body, and byte level. This multi-dimensional mutation approach effectively covers various access control flaws in protected interfaces.

### 6.4. Comparison with Different LLMs

This section evaluates the performance differences between LLMs in firmware code analysis. We compared GPT-4o (a commercial, closed-source model) and Qwen-2.5-Coder-32B (an open-source model), both supporting a maximum context length of 128 K tokens. This selection provides a balanced assessment of our methodology’s generalizability. Additionally, both models excel in analyzing multi-language programming. Notably, Qwen-2.5-Coder-32B ranks first on the “Big Code Models Leaderboard” [57] and has been recognized as the current state-of-the-art (SOTA) open-source code model, matching the coding capabilities of GPT-4o, as highlighted in the “Qwen 2.5-Coder Technical Report” [58].

**Effectiveness of Code Slicing.** The experiments demonstrate that our call-relationship-based slicing strategy substantially enhances the semantic coverage of code analyzable by LLMs. After applying the slicing technique, the total number of analyzable lines increased from 842,953 to 1,274,646, representing a 51.2% expansion. This increase is not merely due to code duplication; while some overlap between slices exists—primarily for shared utility functions invoked across multiple paths—the slicing algorithm employs call-graph pruning and function reuse detection to minimize redundancy. Manual inspection confirmed that the majority of the additional lines correspond to interface-relevant logic that would otherwise be excluded due to LLM context length constraints. Furthermore, we verified that the token lengths of all generated slices remained within the 100K token limit, demonstrating that our slicing strategy can effectively preserve semantic completeness while ensuring compatibility with the LLM’s context window. These results confirm the necessity and effectiveness of slicing: it significantly expands the model’s analyzable scope and enhances the completeness and reliability of downstream vulnerability detection.

**File Information Extraction Comparison.** As shown in Table 5, we systematically evaluated the results of file information extraction from 11 IoT devices using different LLMs. For path information, GPT uniquely extracted 656 paths, both models extracted 894 identical paths, and Qwen uniquely extracted 324 paths. For file identifier information, GPT uniquely extracted 2587 identifiers, both models extracted 8086 identical identifiers, and Qwen uniquely extracted 3833 identifiers.

To further evaluate the actual impact of the extracted file information, we conducted interface probing using the Prober module described in Section 4.3. The Prober sends unauthenticated HTTP requests to each constructed interface and records those with response codes other than 404 as *valid interfaces*. Based on this validation process, GPT uniquely detected 124 valid interfaces, while Qwen detected 1. In total, both models identified 1842 valid interfaces (i.e., with non-404 responses), accounting for 93.6% of all interface candidates constructed from the extracted file information. While Table 5 focuses on static information extraction (paths and identifiers), this additional probing result reflects the effectiveness of information extraction in practice.

These results validate the generality of our approach and demonstrate that the open-source Qwen model effectively supports firmware analysis tasks. Although GPT extracted fewer items than Qwen, it provided higher-quality information extraction with a better valid interface hit rate.

**HTTP Information Extraction Comparison.** As shown in Table 4, ACBreaker(GPT) and ACBreaker(Qwen) showed differences in vulnerability detection, reflecting their varying HTTP information extraction capabilities. ACBreaker(GPT) identified 39 vulnerabilities, while ACBreaker(Qwen) found 33, achieving an 84.6% overall detection rate. Both models effectively support vulnerability detection. However, the gap is evident in parameter manipulation vulnerabilities: ACBreaker(Qwen) detected only 27 of 33, with an 18.2% miss rate, due to inaccuracies in extracting request parameters during code analysis, which affected valid mutation request construction.

While model quality influences extraction accuracy, our method demonstrates strong generality. GPT’s superior parameter extraction enhances detection, especially in precision-sensitive tasks. Qwen, with an 84.6% overall detection rate and 93.6% valid interface identification, shows that open-source models can support ACBreaker effectively. As LLMs improve in code understanding, the intelligent firmware extraction technology will become even more robust.

### 6.5. Case Studies

We now discuss some interesting broken access control vulnerabilities that ACBreaker detected.

#### 6.5.1. Redmi

**Figure 8:**
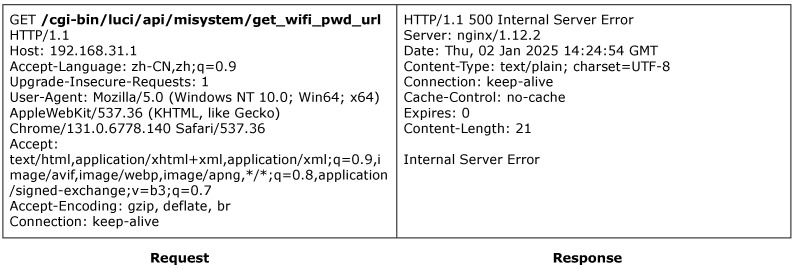
Missing rsa_pubkey parameter: The access to the interface results in a 500 Internal Server Error, indicating that the access control mechanism has blocked the request due to the absence of the necessary parameter validation.

ACBreaker discovered a broken access control vulnerability through parameter manipulation in the Redmi router. The vulnerability exists in the router’s WiFi password query interface /cgi-bin/luci/api/misystem/get_wifi_pwd_url. As shown in Figure 8, accessing this interface without parameters or credentials triggers a 500 Internal Server Error, indicating that the interface is protected.

**Figure 9:**
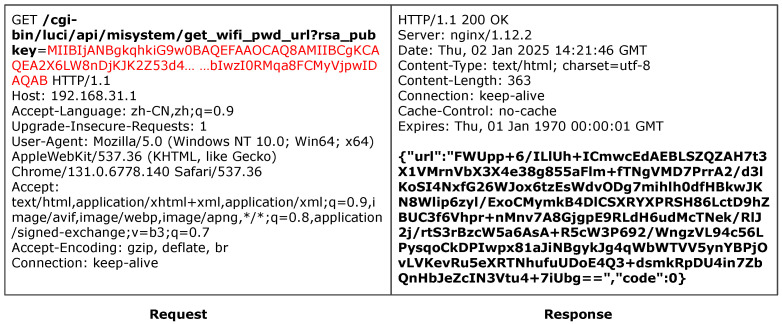
Valid rsa_pubkey parameter: Supplying a random but correctly formatted RSA public key bypasses the access control mechanism, granting unauthorized access to the encrypted WiFi password stored on the device.

This case exemplifies the effectiveness of our LLM-guided two-round inference strategy and the M12 mutation operator. Specifically, ACBreaker first identified the presence of the critical parameter name rsa_pubkey by analyzing the firmware code, which indicated that access to the target functionality requires a valid RSA key. Subsequently, during the second round of inference, the Reasoner analyzed the dynamic value generation logic implemented in Lua scripts and successfully synthesized a correctly formatted rsa_pubkey value. The mutation engine then applied the M12 operator to insert the inferred parameter into the HTTP body, resulting in a valid request, as illustrated in Figure 9. This request successfully bypassed the access control mechanism and retrieved the encrypted WiFi password. Given that IoT devices often reuse management and WiFi passwords, this vulnerability could enable attackers to obtain full administrative control over the device.

This instance demonstrates two critical advantages of our approach. First, the use of multi-step LLM inference enables the discovery of complex dynamic parameters that are not explicitly stored in firmware, overcoming the limitations of static analysis. Second, relying on “parameter hiding” for access control is inadequate, as it embodies the flawed security practice of “security through obscurity”.

#### 6.5.2. D-Link

**Figure 10:**
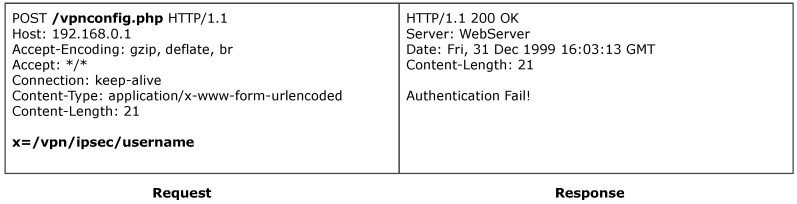
Access with parameters is insufficient: An HTTP request to /vpnconfig.php with required parameters returns a status code 200, but the response content shows “Authentication Fail!”, indicating that a single mutation rule cannot evade the access control mechanism.

**Figure 11:**
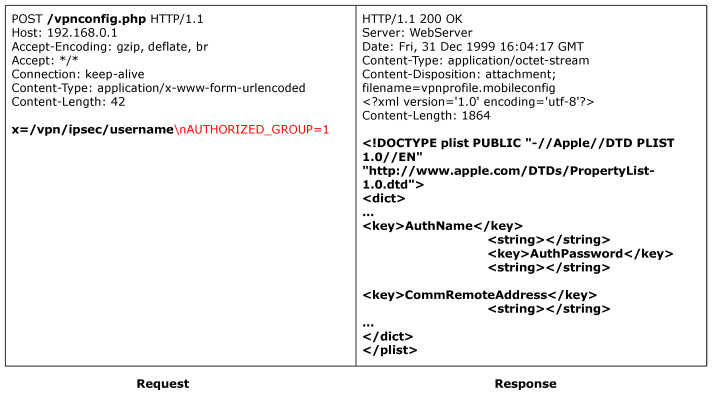
Valid mutation chain: Using two mutation techniques to construct the payload x=/vpn/ipsec/username\nAUTHORIZED_GROUP=1, the device incorrectly parses it, evading the access control mechanism and revealing valid VPN configuration details.

ACBreaker discovered a broken access control vulnerability through parameter manipulation in the D-Link router, which could evade the protected VPN configuration interface /vpnconfig.php. As shown in Figure 10, constructing a request with parameters extracted from intelligence firmware analysis results in an HTTP status code 200, but the response content displays “Authentication Fail!”, indicating that the access control mechanism intercepts unauthenticated requests. To evade this mechanism, ACBreaker combined two mutation rules, parameter injection (M12) and special character mutation (M17), successfully constructing a crafted payload: x=/vpn/ipsec/username\nAUTHORIZED_GROUP=1. As illustrated in Figure 11, submitting this payload causes the cgibin parser to erroneously interpret \nAUTHORIZED_GROUP=1 as a global variable, thereby tampering with the program’s authentication status logic and evade access control checks. This vulnerability uniquely requires both parameter injection and special character handling to be exploited. This case demonstrates two key technical advantages of ACBreaker: first, its comprehensive mutation operators enable effective detection of various types of access control evasion; second, its innovative mutation chain coordination strategy enables the deep exploration of complex vulnerabilities.

#### 6.5.3. TP-Link

**Figure 12:**
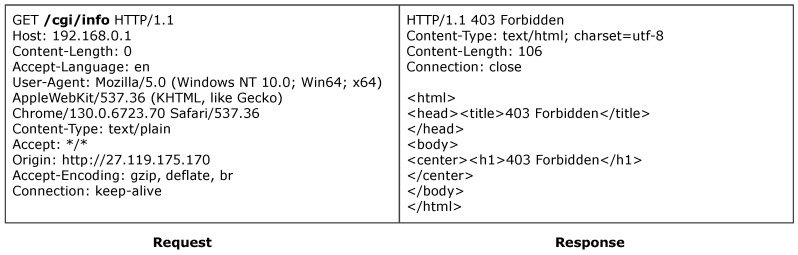
Missing Referer header: Accessing the /cgi/info interface without the critical Referer header results in a 403 Forbidden response, indicating that the access control mechanism blocks unauthenticated requests.

**Figure 13:**
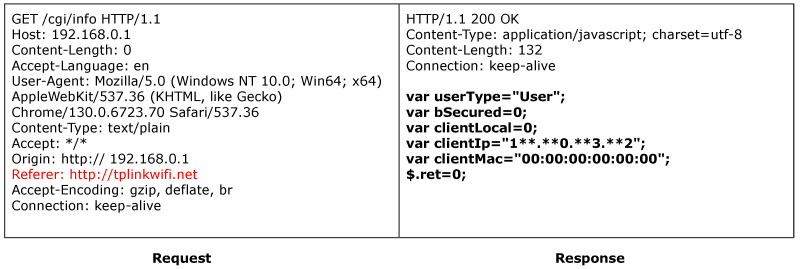
Carrying Referer header: Adding the extracted Referer: http://tplinkwifi.net header to the request evades the access control mechanism, granting access to the device’s configuration information.

ACBreaker discovered a broken access control vulnerability through HTTP header manipulation in the TP-Link router. The vulnerability exists in the router’s device information query interface /cgi/info. As shown in Figure 12, accessing this interface directly results in a 403 Forbidden response, indicating that the interface is protected. During the intelligent firmware analysis phase, ACBreaker extracted the Referer header value “http://tplinkwifi.net”. Based on this information, ACBreaker utilized mutation rule M11 (header injection) to construct an HTTP request carrying this specific Referer value. Subsequently, as illustrated in Figure 13, the mutated request successfully evaded the protected interface and retrieved the device’s configuration information. This case demonstrates two significant findings. First, ACBreaker’s firmware analysis capabilities effectively extract critical validation logic. Second, relying on easily forgeable HTTP headers for access control exemplifies poor security practice, as attackers can arbitrarily modify these headers.

#### 6.5.4. ASUS

**Figure 14:**
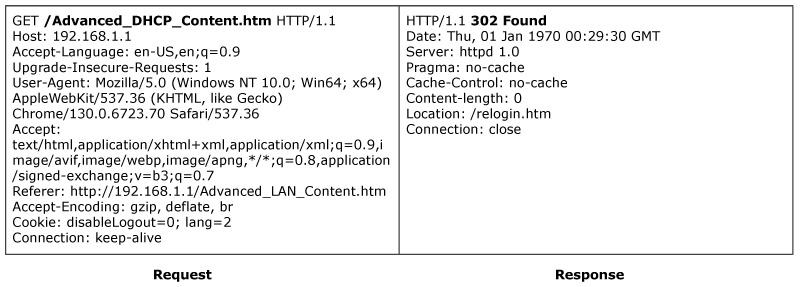
Direct access to protected interface: Accessing the /Advanced_DHCP_Content.htm interface triggers a 302 redirect to the login page /relogin.htm, indicating that the access control mechanism blocks unauthenticated requests.

**Figure 15:**
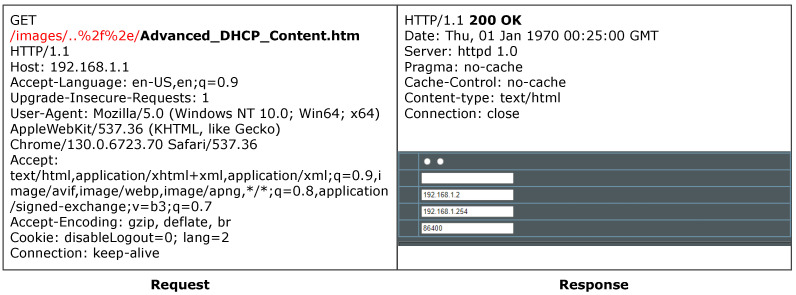
Evasion based on HTTP path manipulation: Using the mutation payload /images/..%2f%2e/Advanced_DHCP_Content.htm, the device evaded the access control mechanism, returning a valid DHCP configuration response.

ACBreaker discovered a broken access control vulnerability through HTTP path manipulation in the ASUS router. The vulnerability exists in the router’s configuration interface /Advanced_DHCP_Content.htm. As shown in Figure 14, accessing this interface directly triggers a 302 redirect to the login page, indicating that the interface is protected. The Prober module of ACBreaker identified that the /images/, /js/, and /lang/ directories were directly accessible without authentication. By combining mutation operators M6 (directory traversal) and M16 (URL encoding transformation), ACBreaker discovered new evasion vectors by combining these directly accessible directories with traversal sequences, such as /js/..%2f%2f and /images/..%2f%2e. As illustrated in Figure 15, these payloads successfully evaded the protected interface, enabling unauthorized access to the device’s DHCP configuration. This case demonstrates two significant findings. First, ACBreaker’s mutation-based approach effectively discovers new evasion vectors beyond known vulnerabilities like CVE-2021-20090 [59]. Second, the vulnerability reveals systematic weaknesses in path normalization implementations, particularly when handling URL-encoded traversal sequences.

## 7. Discussion and Limitations

**Are there ethical concerns in this research?** We prioritized ethical responsibilities and security implications throughout our research. We followed a responsible disclosure process for all broken access control vulnerabilities discovered in this study by promptly reporting these issues to the relevant device manufacturers. To date, Netgear, TP-Link, D-Link, Redmi, and Xiaomi have confirmed these vulnerabilities and assigned CVE identifiers to six affected devices. Furthermore, to ensure the legitimacy of our research, all test devices were commercially purchased through legitimate channels, and testing was conducted strictly within a controlled laboratory environment. We have also confirmed that the manufacturers have patched the vulnerabilities detailed in our case studies. The ACBreaker source code will be released only after all 10 affected devices receive security patches (currently 6/10 patched). Access will be restricted to verified academic researchers through institutional email verification and PI authorization. This controlled release mechanism, combined with technical safeguards against automated exploitation, ensures research reproducibility while preventing potential misuse.

**How to mitigate the security impact of broken access control vulnerabilities on protected interfaces?** Based on our in-depth analysis of access control implementation in IoT device web interfaces, we found that broken access control vulnerabilities in protected interfaces mainly stem from imperfect path parsing mechanisms and inadequate handling of HTTP protocol nuances. At the design level, device manufacturers should abandon the flawed “security through obscurity” practice. In particular, they should avoid relying solely on parameter hiding (e.g., the rsa_pubkey parameter as shown in Section 6.5.1) or on HTTP headers that can be easily forged (e.g., Referer validation as shown in Section 6.5.3) as the primary means of access control. Instead, they should implement strict session-based access control, ensuring that each protected interface undergoes complete authentication and authorization checks.

At the implementation level, devices should establish a unified path normalization processing module to ensure that all URL paths are standardized before being evaluated by the access control mechanisms, particularly focusing on normalizing special cases such as URL-encoded characters and path traversal sequences (e.g., ../as shown in Section 6.5.4). Additionally, developers should strictly adhere to HTTP protocol specifications, performing comprehensive security validation of all request components, including detailed parsing of request methods, headers, and parameters to prevent injection attacks from undermining the integrity of the access control logic. Furthermore, we recommend that manufacturers integrate the ACBreaker into their IoT device development processes. This integration would systematically evaluate web interface access control mechanisms prior to release, thereby enabling early detection and remediation of potential vulnerabilities.

**Are the proposed mutation strategies sufficiently comprehensive?** When designing ACBreaker, our research scope focused on fuzzing access control mechanisms in protected interfaces based on the HTTP protocol. This scope selection was based on two key factors: first, HTTP is the primary protocol for IoT device web management interfaces [60]; second, HTTP-based access control typically functions separately from other authentication mechanisms (e.g., NFC, Bluetooth handshakes, or cloud-based OAuth). Our experimental evaluation confirmed the effectiveness of this approach in discovering broken access control vulnerabilities within this scope. Although our mutation operators primarily target HTTP-based interface access control mechanisms, the underlying design principles are broadly applicable and can be extended to other protocols as needed. Security researchers can develop and add new mutation operators based on specific testing requirements while maintaining the same objectives, thereby enhancing the efficiency of access control security testing for protected interfaces.

**Why use code slicing instead of RAG technology?** In handling firmware code that exceeds the LLM context window, we evaluated two technical approaches: a slicing strategy based on call relationships and Retrieval-Augmented Generation (RAG), the latter of which utilizes vectorized code data to supplement the context of the LLM. We ultimately chose the call-relationship-based slicing strategy based on three considerations: First, the function call relationships, as the core skeleton of program logic flow, effectively preserves the structural characteristics of the interface-related code. Second, this lightweight slicing method efficiently handles multi-language hybrid code in IoT firmware. Finally, current RAG technology shows significant limitations in processing complex code semantics and cross-file dependencies [61,62,63,64], particularly in maintaining completeness when retrieving parameter parsing and access control logic related to web interfaces, which could lead to missed potential broken access control vulnerabilities. In contrast, the call-relationship-based slicing strategy systematically preserves complete call chains of interface processing, making it more suitable for vulnerability detection scenarios in this research. However, we also recognize that the call-relationship-based slicing strategy has limitations in handling configuration file information, which may lead to incomplete parameter constraint extraction and potentially cause false negatives. In the future, we plan to explore solutions that integrate configuration file analysis with code slicing to enhance our capability to extract parameter constraint information.

**Future Work.** This research conducts an in-depth analysis of access control bypass vulnerabilities in IoT device interfaces based on HTTP protocols, representing only one aspect of IoT security. Future research could extend to other widely used IoT protocols, such as MQTT and UPnP, to comprehensively evaluate the security of IoT devices across different communication protocols. Meanwhile, this research demonstrates the potential of large language models in analyzing heterogeneous firmware code. This capability can be further applied to assist in detecting other types of vulnerabilities in IoT firmware, such as command injection and SQL injection vulnerabilities. However, using this methodology to a broader range of IoT devices may encounter challenges due to the heterogeneity of device architectures, firmware types, and communication protocols. Moreover, combined with the latest advances in automatic vulnerability repair, such as the SKYPORT framework proposed by Youkun Shi et al. [65], future work could automatically generate corresponding security patches after identifying access control vulnerabilities, thereby achieving a closed-loop process from vulnerability discovery to repair. This would not only improve the efficiency of security fixes for IoT devices but also provide manufacturers with more operational security solutions, ultimately promoting the overall security level of the IoT ecosystem.

## 8. Conclusions

In this paper, we present ACBreaker, a novel automated tool designed to detect broken access control vulnerabilities in the protected interfaces of IoT devices. This tool leverages the code-understanding capabilities of the LLM to extract device-specific information from heterogeneous firmware, thereby constraining the fuzzing space. It employs 17 carefully designed mutation operators to generate effective mutation requests. In the evaluation of 11 mainstream IoT devices, ACBreaker analyzed 1,274,646 lines of heterogeneous code, successfully discovering 39 previously unknown vulnerabilities, which affect a total of 508 protected interfaces. These vulnerabilities were categorized into three typical types: HTTP path manipulation, parameter manipulation, and HTTP header manipulation. We have responsibly disclosed all identified vulnerabilities to the affected device manufacturers, with six IoT devices having received CVE IDs and having been fixed. We hope this study will encourage the community to collectively address the growing security threats related to access control in IoT device web interfaces.

## Figures and Tables

**Table 1:** Overview of mutation operations and payload examples (mutations marked with a star (*) indicate rules also implemented in the open-source tool nomore403).

Target	OP	Payload Example		Description
		Original	Mutation	
Request Line	M1 *	GET	HEAD	Variations of HTTP request methods.
	M2 *	HTTP /1.1	HTTP /0.9	Variations of HTTP request protocol versions.
	M3	/admin/	/;/admin/	Prefix insertion mutations applied to HTTP paths.
	M4 *	/admin	/admin?x=1.jpg	Suffix insertion mutations applied to HTTP paths.
	M5 *	/admin/index	/admin;a=b/index	Hierarchical insertion mutations applied to HTTP paths.
	M6	/admin	/static/../admin	Path mutations based on permissible access paths.
	M7	/admin	/admin?public.cgi	Path mutations based on permissible files.
	M8	/admin/main.aspx	/admin/(S(X))/main.aspx	Exploitation of ASP.NET cookieless session features.
Headers	M9 *	<None>	X-Remote-IP: 127.0.0.1	Insertion of IP-related HTTP headers to bypass IP-based access controls.
	M10	<None>	X-Rewrite-URL: /admin	Insertion of HTTP headers potentially affecting server path parsing.
	M11	<None>	Referer: tplinklogin.net	Insertion of headers and corresponding values extracted from firmware.
Body	M12	<None>	param=value	Insertion of parameters and their inferred values extracted from firmware.
Byte	M13	<None>	%00	Injection of special characters (e.g., control characters).
	M14 *	/admin	/Admin	Mutation by altering the case of characters.
	M15	/aDmin	/aDmin	Homoglyph substitution using Unicode lookalike characters.
	M16 *	/admin	/a%64min	URL encoding, including multiple levels of encoding.
	M17	/system.php?public.cgi	/system.php%00public.cgi	Replacing special characters (e.g., &, ?, #) with %00 or control characters.

**Table 2:** Overview of evaluated IoT devices.

Device ID	Vendor	Model	Device Type	Firmware Version	Web Type	Web Code LoC
1	Netgear	DGN2200	Modem router	V1.0.0.46_7.0.44	Bin+HTML	63,960
2	Netgear	WNR614	WiFi router	V1.1.0.28_1.0.1WW	Bin+HTML	95,588
3	Netgear	WNDR3700 v1	WiFi router	V1.0.16.98	Bin+HTML	68,680
4	TP-Link	TL-WR840N v6	VPN router	0.9.1 4.16	Bin+HTML	19,685
5	TP-Link	Archer C20	WiFi router	V6.6_230412	Bin+HTML	38,373
6	D-Link	DIR-822	VPN router	1.03	Bin+PHP	118,851
7	D-Link	DIR-859	WiFi router	A3 1.05	Bin+PHP+HTML	102,198
8	D-Link	DNS-320L	NAS	A3 1.03	Bin+PHP+HTML	289,766
9	Redmi	AX1800	WiFi router	1.0.88	Bin+LUA+HTML	183,554
10	Xiaomi	AC2100	VPN router	2.0.743	Bin+LUA+HTML	171,627
11	ASUS	DSL-AC88U	Modem router	v1.10.05_build502	Bin+ASP+HTML	122,364

**Table 3:** Summary of broken access control vulnerabilities discovered by ACBreaker across IoT devices.

Device ID	#Hints	#Vuln Nums	Vuln Type	CVE Status
1	257	2	HTTP Path Manipulation	Assigned
2	83	2	HTTP Path Manipulation	Pending Assignment
3	0	0	None	NoVuln
4	2	1	HTTP Header Manipulation	Assigned
5	2	1	HTTP Header Manipulation	Assigned
6	23	23	Parameter Manipulation	Pending Assignment
7	3	3	Parameter Manipulation	Assigned
8	1	1	Parameter Manipulation	Pending Assignment
9	2	2	Parameter Manipulation	Assigned
10	2	2	Parameter Manipulation	Assigned
11	133	2	HTTP Path Manipulation	Pending Assignment
**Total**	**508**	**39**	-	-

**Table 4:** Comparison of broken access control vulnerability detection across different implementations. The numbers in parentheses represent the number of protected interfaces affected by the vulnerability.

Device ID	#Valid	Interfaces with Broken Access Control Vulnerabilities				
		nomore403	BooFuzz	ACBreaker (noalg)	ACBreaker (Qwen)	ACBreaker (GPT)
1	302	0 (0)	0 (0)	2 (42)	2 (257)	2 (257)
2	365	0 (0)	0 (0)	2 (17)	2 (79)	2 (83)
3	149	0 (0)	0 (0)	0 (0)	0 (0)	0 (0)
4	115	2 (2)	0 (0)	1 (1)	1 (2)	1 (2)
5	87	2 (2)	0 (0)	1 (2)	1 (2)	1 (2)
6	119	0 (0)	0 (0)	14 (14)	21 (21)	23 (23)
7	78	0 (0)	0 (0)	2 (2)	3 (3)	3 (3)
8	217	0 (0)	0 (0)	1 (1)	1 (1)	1 (1)
9	240	0 (0)	0 (0)	1 (1)	0 (0)	2 (2)
10	158	0 (0)	0 (0)	2 (2)	0 (0)	2 (2)
11	137	0 (0)	0 (0)	2 (22)	2 (133)	2 (133)
**Total**	**1967**	**4 (4)**	**0 (0)**	**28 (104)**	**33 (498)**	**39 (508)**

Green indicates the best performance regarding the number of vulnerabilities or affected interfaces among all tools.

**Table 5:** Comparison of web interface information extraction between GPT and Qwen across 11 IoT devices.

Device ID	Path			File Identifier			Valid Interfaces		
	GPT Only	Both	Qwen Only	GPT Only	Both	Qwen Only	GPT Only	Both	Qwen Only
1	20	21	7	145	538	131	20	282	0
2	20	30	6	196	545	120	24	341	0
3	6	22	18	184	950	727	0	149	0
4	20	22	6	145	538	131	1	114	0
5	20	19	30	130	330	58	2	85	0
6	213	236	81	414	1399	891	0	119	0
7	166	192	50	289	1249	617	3	75	0
8	74	136	34	238	890	293	44	173	0
9	49	90	42	275	588	397	2	238	0
10	58	99	43	477	792	318	27	130	1
11	10	27	7	94	267	150	1	136	0
Total	656	894	324	2587	8086	3833	124	1528	1

This table presents the comparison of information extraction capabilities between GPT-4o and Qwen-2.5-Coder-32B across three dimensions: path, file identifier, and valid interfaces. For each dimension, **GPT only** indicates the number of items extracted exclusively by GPT, **Both** indicates the number of items extracted by both models, and **Qwen only** indicates items extracted exclusively by Qwen. The analysis covers 11 IoT devices, with Device ID indicating each unique device in our dataset. Green highlights cases where GPT performs better than Qwen. Orange highlights cases where Qwen extracts more items than GPT.

## Data Availability

Available upon request.

## Abbreviations

The following abbreviations are used in this manuscript:

EWA	Embedded Web Application
IoT	Internet of Things
CVE	Common Vulnerabilities and Exposures
CGI	Common Gateway Interface
GPT	Generative Pre-trained Transformer
RAG	Retrieval-Augmented Generation
LLM	Large Language Model
CoT	Chain-of-Thought
HTTP	HyperText Transfer Protocol
RAG	Retrieval-Augmented Generation
